# (Disparity-Driven) Accommodation Response Contributes to Perceived Depth

**DOI:** 10.3389/fnins.2018.00973

**Published:** 2018-12-18

**Authors:** Cyril Vienne, Justin Plantier, Pascaline Neveu, Anne-Emmanuelle Priot

**Affiliations:** Institut de Recherche Biomédicale des Armées, Bretigny sur Orge, France

**Keywords:** accommodation, vergence, oculomotor cues, perceived depth, sensory conflict, stereoscopic displays

## Abstract

When looking at objects at various distances in the physical space, the accommodation and vergence systems adjust their parameters to provide a single and clear vision of the world. Subtended muscle activity provides oculomotor cues that can contribute to the perception of depth and distance. While several studies have outlined the role of vergence in distance perception, little is known about the contribution of its concommitant accommodation component. It is possible to unravel the role of each of these physiological systems by placing observers in a situation where there is a conflict between accommodation and vergence distances. We thus sought to determine the contribution of each response system to perceived depth by simultaneously measuring vergence and accommodation while participants judged the depth of 3D stimuli. The distance conflict decreased depth constancy for stimulus displayed with negative disparity steps (divergence). Although vergence was unaffected by the stimulus distance, accommodation responses were significantly reduced when the stimulus was displayed with negative disparities. Our results show that biases in perceived depth follow undershoots in the disparity-driven accommodation response. These findings suggest that accommodation responses (i.e., from oculomotor information) can contribute to perceived depth.

## 1. Introduction

Many visual cues can contribute to space perception. They can either be retinal, including all geometrical patterns cast onto retinas (e.g., perspective, occlusion, optic flow) or extra-retinal, subtending information that is not imaged on the back of the eyes (e.g., oculomotor activity). Gauthier et al. (1990) found, for example, by exerting a passive deviation on one occluded eye, that proprioception affected spatial localization by the viewing eye, thereby showing the role of afference in position coding. Oculomotor cues not only arise from sensing these proprioceptive inputs, but also from sensing the motor commands to the ocular muscles (Bridgeman and Stark, 1991). As such, motor efference and/or proprioception from the vergence system are considered as sources of information for distance perception (Brenner and Van Damme, 1998). It was found that changes in vergence state using prismatic deviation consistently altered the way observers judged distance (Tresilian et al., 1999; Priot et al., 2012). Studies typically suggest that oculomotor cues to distance are only efficient in the near space, and notably in impoverished scenes (Kunnapas, 1968; Tresilian et al., 1999). However, recent empirical evidence has revealed the importance of cues from focus (i.e., accommodation and blur cues) and vergence in perceiving depth in stereoscopic displays. We thus sought to determine the contribution of each response system to perceived depth. In this study, we describe results that suggest accommodation - from disparity-driven accommodation–as an important source of bias in perceived depth.

To provide a clear vision of the world, the accommodation system minimizes blur on the retinal image by changing the shape of the crystalline lens with respect to the retinae. In principle, accommodation is the source of two types of distance information: retinal blur and motor activity. However, the contribution of accommodation to perceived distance is controversial (Mon-Williams and Tresilian, 2000). When accommodation is considered alone, though estimates covary with stimulus distances, they are highly variable, and notable inter-individual differences have been found (Wallach and Norris, 1963; Fisher and Ciuffreda, 1988; Mon-Williams and Tresilian, 2000). Consequently, it has been suggested that rather than providing absolute distance information, accommodation may only serve as an ordinal cue to depth, potentially conveyed through accommodation-vergence interactions (Mon-Williams and Tresilian, 2000). More recent studies have suggested that both accommodation and blur may provide a more useful cue to depth than expected (Watt et al., 2005; Held et al., 2012). Some experimental studies reported reduced depth constancy when the stimulus was displayed with conflicting accommodation and vergence distances, such that depth perception was biased toward the screen plane distance (Watt et al., 2005; Hoffman et al., 2008; Vienne et al., 2015). The reported bias could not be attributed to the accommodative response (i.e., extra-retinal signal) or the accommodative stimulus (i.e., retinal blur) because accommodation responses were not measured. Though defocus blur is known to affect perceived depth (e.g., in photographs Held et al., 2010), neural commands for accommodation (Hoffman et al., 2008) as well as proprioception (Held et al., 2012) have also been proposed as sources of distance information from accommodation. Therefore, the purpose of the current study was to investigate whether accommodation response, as opposed to blur, influences perceived depth, by simultaneously measuring accommodation and vergence responses while observers judged 3D stimuli.

The individual influences of vergence and accommodation on perceived depth are difficult to unravel owing to their mutual interactions (Schor, 1992). The two systems can act upon each other through neural crosslinks. A vergence response can be produced by the presentation of blur on the retinal image (stimulus-to-accommodation), which is called the blur-driven vergence component. Similarly, an accommodation response can follow the presentation of binocular disparity (stimulus-to-vergence), which is called the disparity-driven accommodation response. The latter allows more rapid accommodation, as vergence has faster timing dynamics. Thus, the shape and timing of the final responses of both systems are influenced by primary cues, neural cross-links and negative feedback controlling error. One way to overcome this cross-dependence is to employ a cue conflict paradigm in which the vergence distance and the accommodation distance are set to different values. Using such a conflict paradigm, Vienne et al. (2014) revealed that vergence was significantly affected by the absence of blur cues; responses were slower with longer latency. The authors also found that the accommodation-vergence conflict had more impact on divergence latency than convergence latency. Such differences between convergence and divergence, or between accommodation and disaccommodation (i.e., a decrease in accommodation), can be accounted for by the existence of two, separate neurophysiological systems with different dynamic properties (Alvarez et al., 2005; Schor and Bharadwaj, 2006). Consequently, presenting conflicting accommodation and vergence distances with a fixed focal distance may result in differential effects on accommodation and disaccommodation responses.

The cue conflict paradigm used in the present study was based on fixed focal distances, while vergence was stimulated by positive (convergent) or negative (divergent) steps; as a result, accommodation was driven by binocular disparity–though we cannot totally exclude a potential influence of the proximal component. It is worth noting that binocular disparity can drive accommodation in a way that is similar to retinal blur, and their equivalent dynamic characteristics suggest that shared neural pathways control the two systems (Suryakumar et al., 2007). If accommodation responses contribute to depth perception, then they should be influenced by the accommodation-vergence conflict as much as depth perception was shown to be affected in previous studies. Our results reveal that the accommodation-vergence distance conflict influences both the perceptual estimation and the accommodation response for negative disparity steps.

## 2. Method

### 2.1. Participants

Fourteen observers (9 males) were recruited for this study. They were 36.9 years old on average (23, 25, 27, 31, 33, 34, 36, 37, 41, 42, 42, 46, 47, and 57 years old). All had normal or corrected to normal vision and presented a stereoacuity threshold < 60 arcmin, as assessed by the TNO Test. All subjects provided informed consent prior to the experiment. The oculomotor responses of one participant (the one that were 57 years old) could not be analyzed because his pupil sizes were too small for the refractometer to work correctly. It is worth noting that no participants did complaint about difficulties focusing at the near distance manipulated in this experiment. However, because of the age range of the participants and the potential influence of presbyopia on accommodation amplitude (Duane, 1922), an analysis of accommodation responses as a function of age, which revealed no difficulties for the older participants with the near distances employed in this experiment, is provided below (see Appendix).

### 2.2. Apparatus

A 3DLP video-projector (Christie Mirage WU7, 1920 1,200 pixels, 7,000 Lumens) displayed the stimuli on a projection screen (200 × 150 cm maximal size, ORAY). The black level was about 1 cd.ma2 measured using a spectroradiometer (Minolta CS1000). The participants wore 120 Hz active shutter glasses (Volfoni) to fuse the left and right views. Stimuli were designed in OpenGL and were displayed using the PsychToolbox extension for MATLAB (Brainard, 1997). Stereoviews were designed according to an off-axis rendering method (Vienne et al., 2016). The inter-axial separation between the left and right simulated cameras was adjusted according to each individual inter-ocular distance measured with a Pupil Distance Meter (PD-82II, Towal Medical Instruments). Accommodation and vergence responses were measured using a commercially available refractometer (PlusOptix PowerRef II, 25 Hz) placed to the right of the participant. A large beam splitter was oriented at 45 degrees and placed in front of the observer, deviating infrared light emanating from the refractometer so that rays could reach the participant's eyes. A chin rest was used to maintain the participant's head. To vary both the accommodation distance and the vergence distance, a specific device was designed. Wooden rails were fastened on the floor to move the screen plane and the projector that was set on a rolling support. The projector was also moved to provide the same angular display size across viewing conditions.

### 2.3. Task and Stimulus

**Figure 1:**
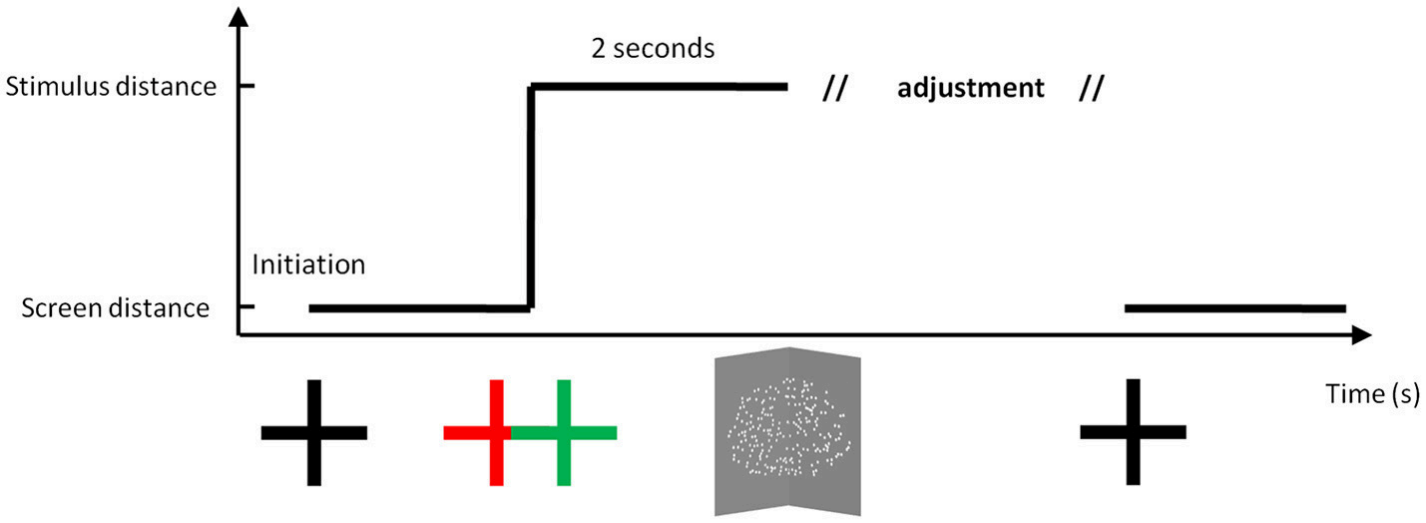
A typical trial. Before initiation, the fixation cross was located on the screen plane at a given distance (57, 80, and 133 cm). Following initiation by the participant, the fixation cross jumped in depth or remained in the same plane, and then stayed on the display for 2 s. The apex of the angle was then placed at one of three possible distances (57, 80, and 133 cm), while the screen plane was set at one of these three possible distances. After adjusting the hinge angle, the cross returned back to the screen plane. Drawing is not to scale.

Figure 1 shows the temporal series of a typical trial. The participant was asked to initially fixate a white cross, while limiting the occurrence of blinks. At trial initiation, they could choose when to press a button that changed the distance of the cross (or not). At this time, the fixation target could jump into depth either behind or in front of the screen plane, or could stay at the same depth. After initiation, the cross remained for 2 s before a cloud of dots appeared. When stereo-views were fused, the cloud of dots depicted a vertical dihedral angle. The participant was asked to maintain fixation on the stimulus, and adjust the angle to make it correspond to 90 degrees using a response box. When satisfied with the adjustment, the participant validated their decision and the fixation cross returned back to its initial distance in the screen plane.

The stimulus was composed of white dots over a gray background forming an elliptical aperture 14 degrees wide and 7 degrees high. Dot density was about 1 dot per degree. There were three accommodation distances and three vergence distances (i.e., 0.57, 0.80, and 1.33 m) making a total of nine measurement groups, with ten trials in each group. Conflict size was either 0, 0.5, or 1 Diopter.

### 2.4. Data Analysis

Angle adjustments were converted into scaling distances, i.e., the distance at which the horizontal disparities in the stimulus specified a right angle. For a target straight ahead of the viewer, the scaling distance SD is

$$\begin{array}{r} SD=\frac{IOD\left(HSR+1\right)}{2\left(HSR-1\right)}tan\left(-\pi /4\right) \end{array}$$

where IOD is the inter-ocular distance and HSR is the horizontal size ratio, a measure of relative horizontal disparity (Howard, 2002). Uncertainty was measured as standard deviation over ten depth estimates in each measurement group for each individual. Before recording accommodation data, a calibration step was performed for each participant at the beginning of the experiment. In doing so, the right eye was occluded with a Wratten filter while the left eye fixated a Snellen letter at 80 cm. During fixation with the left eye, trial lenses (–4 D to +4 D in 1-D step) were placed in front of the right occluded eye. A correction factor was computed from a linear regression performed between measured and expected refraction and applied to accommodation responses. This correction factor was applied to the accommodation responses. To analyze accommodation and vergence responses, data were filtered using a moving average over five data samples. Vergence was computed as left-eye position minus right-eye position. Accommodation and vergence responses were measured as the averaged amplitude (i.e., the difference between the target position minus the baseline) over the last second preceding the appearance of the diheadral angle (i.e., during the last second of cross fixation).

Statistical analyses were performed using the *afex* package (Singmann et al., 2016) in R software (R Core Team, 2013) and thanks to custom Matlab scripts.

## 3. Results

### 3.1. Perceptual Adjustments

**Figure 2:**
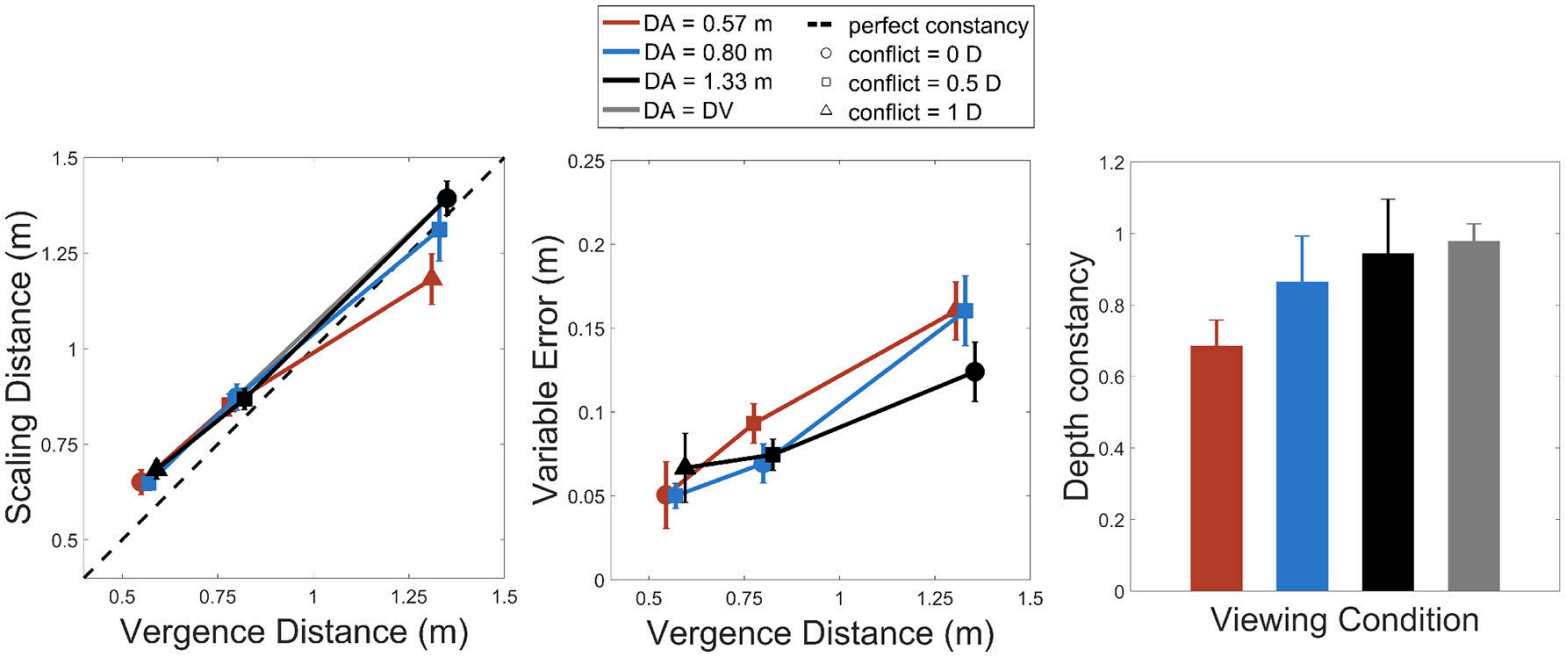
**Left**: scaling distances as a function of vergence distance for the three accommodation distances (red: 57 cm, blue: 80 cm, and black: 133 cm). **Middle**: variable error as a function of vergence distance for the same conditions. **Right**: slopes of depth constancy as a function of accommodation distances. The gray bar represents the condition where accommodation distance equals vergence distance. Vertical error bars show 95% Cousineau-Morey confidence intervals for within-subjects designs (Cousineau, 2005; Morey, 2008).

Figure 2 summarizes the results obtained for the depth adjustment task, both for scaling distances and variable error in depth estimation. The left chart of Figure 2 represents how scaling distance varies with vergence distance for the three accommodation distances (*D*_*A*_ = 0.57 m, *D*_*A*_ = 0.8 m, and *D*_*A*_ = 1.33 m). ANOVAs revealed a statistically significant effect of accommodation distance on scaling distance, *F*_(_2, 26) = 8.19, *p* < 0.002, with sphericity corrected using Greenhouse-Geisser (GG) estimate: F(1.25, 16.31)= 8.19, *p* < 0.008, as well as an effect of vergence distance [*F*_(2, 26)_ = 165.19, *p* < 0.0001, with sphericity corrected using Greenhouse-Geisser (GG) estimate: *F*_(1.02, 13.29)_ = 165.19, *p* < 0.0001]. The analysis revealed an interaction effect between the accommodation and the vergence distances on the scaling distances [*F*_(4, 52)_ = 6.78, *p* < 0.0002, with sphericity corrected using the GG estimate: *F*_(1.65, 21.42)_ = 6.78, *p* < 0.008). A *post-hoc* analysis revealed that, for the farthest vergence distance (*D*_*V*_ = 1.33 m), the scaling distances for the near accommodation distance (*D*_*A*_ = 0.57 m, in red) were smaller than for the two other accommodation distances (vs. *D*_*A*_ = 0.8 m, in blue, and vs. *D*_*A*_ = 1.33 m, in black, *p* < 0.003, *p* < 0.0001, respectively).

The middle chart of Figure 2 represents how variable error changes with vergence distance for the three accommodation distances. The analysis performed on variable error revealed an interaction effect between the accommodation and the vergence distances [*F*_(4, 52)_ = 4.05, *p* < 0.007], a significant effect of vergence distance [*F*_(2, 26)_ = 138.1, *p* < 0.0001, with sphericity corrected using Greenhouse-Geisser (GG) estimate: [*F*_(1.42, 18.48)_ = 138.1, *p* < 0.0.0001] but no effect of accommodation distance [*F*_(2, 26)_ = 1.93, *p* > 0.05). The variable error thus increased with increasing viewing distance and this error tended to increase more strongly for near accommodation distances.

The right chart of Figure 2 represents slopes of depth constancy (slopes of linear regression between scaling distances and vergence distances for each accommodation distance) for the different viewing conditions (the three accommodation distances and the condition where accommodation and vergence distances matched). Analyses on depth constancy slopes reveal an effect of viewing condition (slopes for *D*_*A*_ = 0.57*m*, for *D*_*A*_ = 0.8*m*, for *D*_*A*_ = 1.33*m* and for slopes where *D*_*A*_ = *D*_*V*_) [*F*_(3, 39)_ = 10.87, *p* < 0.0001, with sphericity corrected using the GG estimate: [*F*_(1.53, 19.9)_ = 10.87, *p* < 0.002). Pairwise *t*-tests with Bonferroni corrections reveal that the slope for the near accommodation distance (*D*_*A*_ = 0.57 m, in red) was lower than the ones for the farther accommodation distances (*D*_*A*_ = 0.8 m and *D*_*A*_ = 1.33 m) and matched accommodation-vergence distances (*p* < 0.02, *p* < 0.0003, *p* < 0.0001).

### 3.2. Accommodation and Vergence Responses

**Figure 3:**
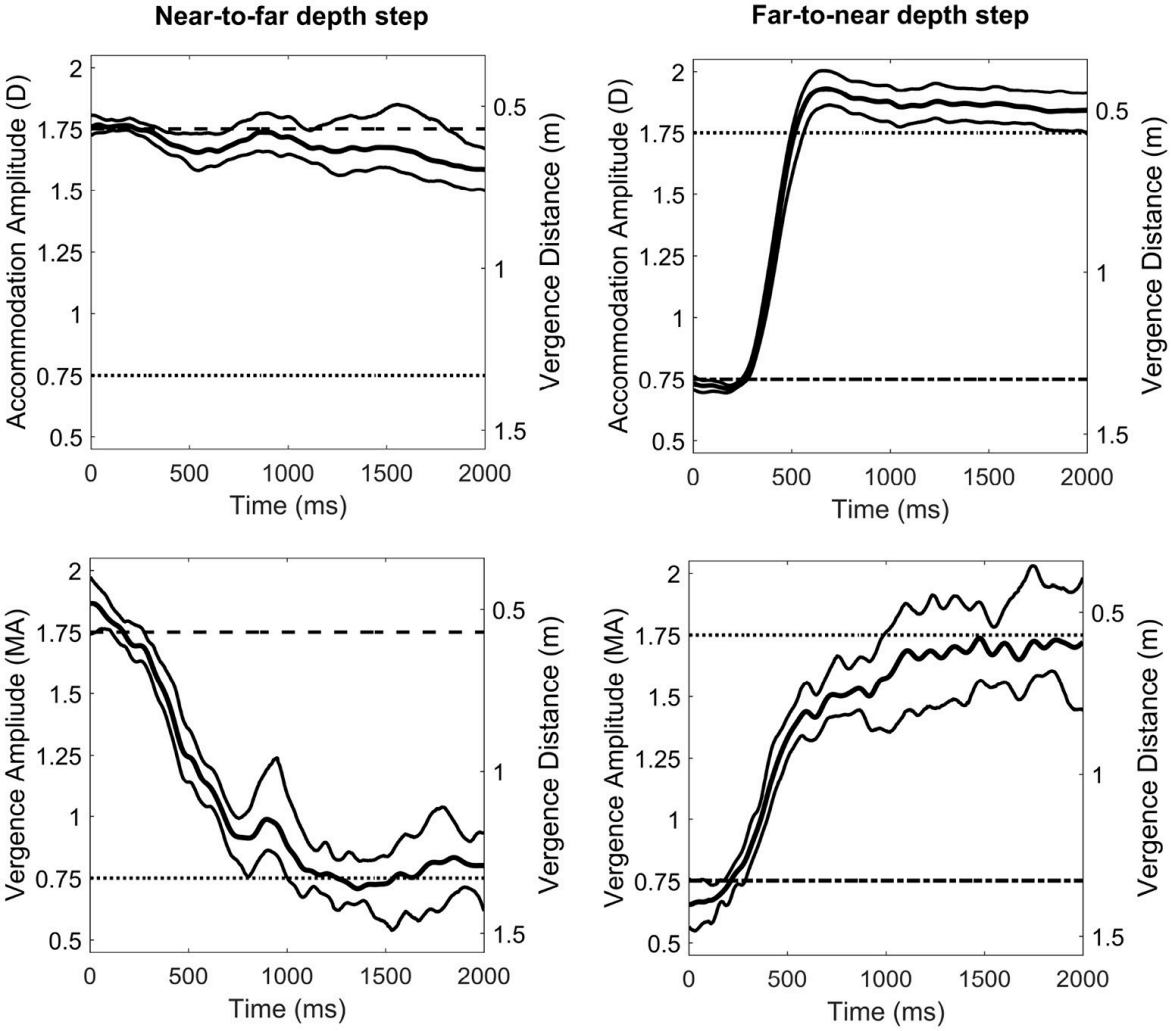
Averaged accommodation **(Top)** and vergence **(Bottom)** responses to a ± 1 Diopter depth step of a representative observer. The left column shows near-to-far depth steps (i.e., disaccommodation and divergence) and the right column shows far-to-near depth steps (i.e., accommodation and convergence). D stands for Diopters and MA for Meter Angles. Central curves depict the averaged response over ten trials. Thin curves indicate the 95% bootstrap confidence interval of the mean response.

**Figure 4:**
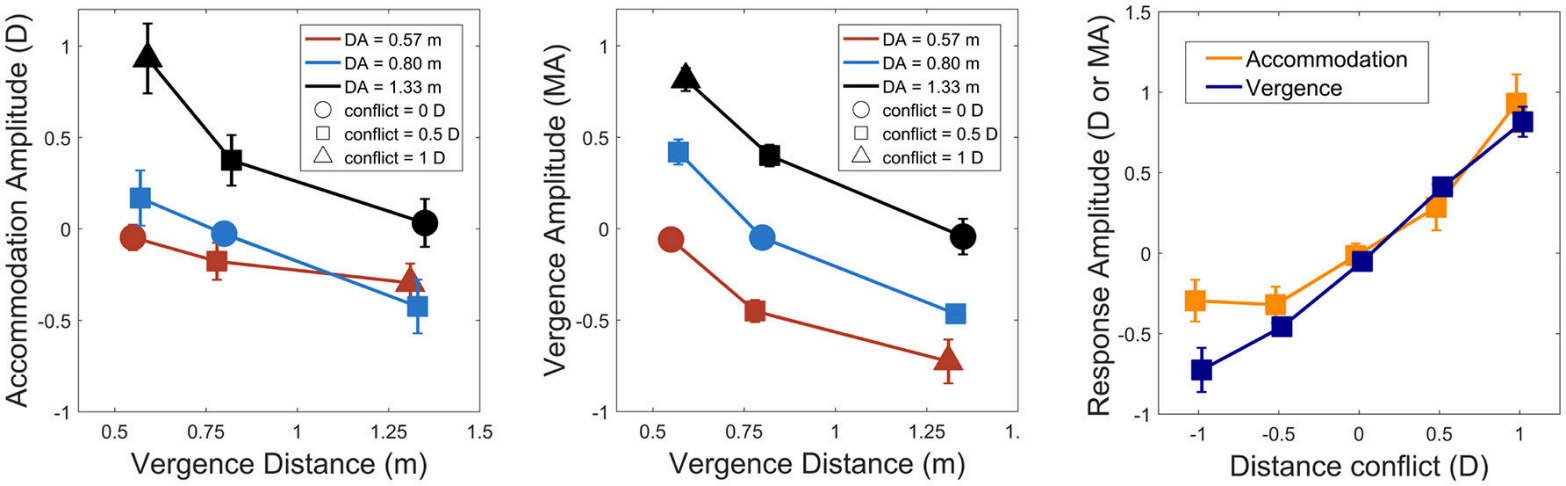
**Left**: accommodation response as a function of vergence distance, accommodation distance (red: 0.57 m, blue: 0.8 m and black: 1.33 m, circle: no conflict) and distance conflict (square: ± 0.5 D, triangle: ± 1 D). **Middle**: vergence response as a function of vergence distance, accommodation distance, and distance conflict (same legend). Negative responses indicate disaccommodation or divergence response functions whereas positive responses indicate accommodation or convergence response steps. **Right**: accommodation and vergence response amplitude as a function of distance conflict. Accommodation is depicted in dark orange and vergence in dark blue. Vertical error bars show 95% Cousineau-Morey confidence intervals for within-subjects designs (Cousineau, 2005; Morey, 2008).

Figure 3 shows sample accommodation and vergence responses for a representative observer. We observed a consistent undershoot of disaccommodation responses for negative steps (i.e., smaller amplitudes than expected) whereas the gain of accommodation responses was widely better for positive steps. However, for vergence, whatever the direction of the oculomotor response (i.e., near-to-far depth steps or far-to-near depth steps), the gain was nearly around 1, suggesting that the vergence system could respond to binocular disparity and that the possible contribution of the blur-driven component of vergence was modest. Accommodation and vergence responses were analyzed by computing response amplitudes for each possible combination of accommodation and vergence distances for comparison with the perceptual estimations observed above. Figure 4 represents accommodative and vergence amplitudes as a function of vergence distance and accommodation distance. A two-way repeated measures ANOVA performed on accommodation amplitude revealed a significant effect of accommodation distance [*F*_(2, 24)_ = 67.15, *p* < 0.0001], a significant effect of vergence distance [*F*_(2, 24)_ = 58.7, *p*>0.0001], and an interaction effect between the two [*F*_(4, 48)_ = 7.62, *p* < 0.0001, with sphericity corrected using the GG estimate: *F*_(2.08, 25)_ = 7.62, *p* < 0.0001]. The effect of vergence distance was smaller for the nearer accommodation distance (*D*_*A*_ = 0.57 m) than for the two other distances. These results are depicted in Figure 4 (Left). The same analysis was performed on vergence amplitude and revealed a significant effect of accommodation distance [*F*_(2, 24)_ =314.6, *p* < 0.0001, with sphericity corrected using the GG estimate: [*F*_(1.33, 16)_ = 314.6, *p* < 0.0001], a significant effect of vergence distance [*F*_(2, 24)_ = 331.1, *p* < 0.0001], and a small interaction effect between the two [*F*_(4, 48)_ = 3.05, *p* < 0.05] (see Figure 4, middle).

To compare both systems, accommodation and vergence responses were then analyzed by computing their response amplitudes for each possible distance conflict (–1, –0.5, 0.5, 1, and the no conflict condition). Conflict size was computed as $C=\frac{1}{D_{V}}-\frac{1}{D_{A}}$, *D*_*V*_ stands for vergence distance and *D*_*A*_ stands for accommodation distance. A two-way repeated measures ANOVA revealed a significant difference between accommodation and vergence on response amplitude [*F*_(1, 12)_ = 13.06, *p* < 0.01). The analysis also revealed an effect of the conflict on response amplitude [*F*_(4, 48)_ = 199.87, *p* < 0.0001, with sphericity corrected using the GG estimate: [*F*_(2.7, 31.9)_ = 199.87, *p* < 0.0001]. An interaction effect between the two variables was also observed [*F*_(4, 48)_ = 7.59, *p* < 0.0001, with sphericity corrected using the GG estimate: [*F*_(2.3, 27.8)_ = 7.59, *p* < 0.001]. A *post-hoc* analysis revealed that accommodation and vergence amplitudes only differed significantly when the conflict was -1 Diopter (divergent step, see Figure 4, right).

### 3.3. Contribution of Accommodation and Vergence Response Amplitudes to Perceived Depth

**Figure 5:**
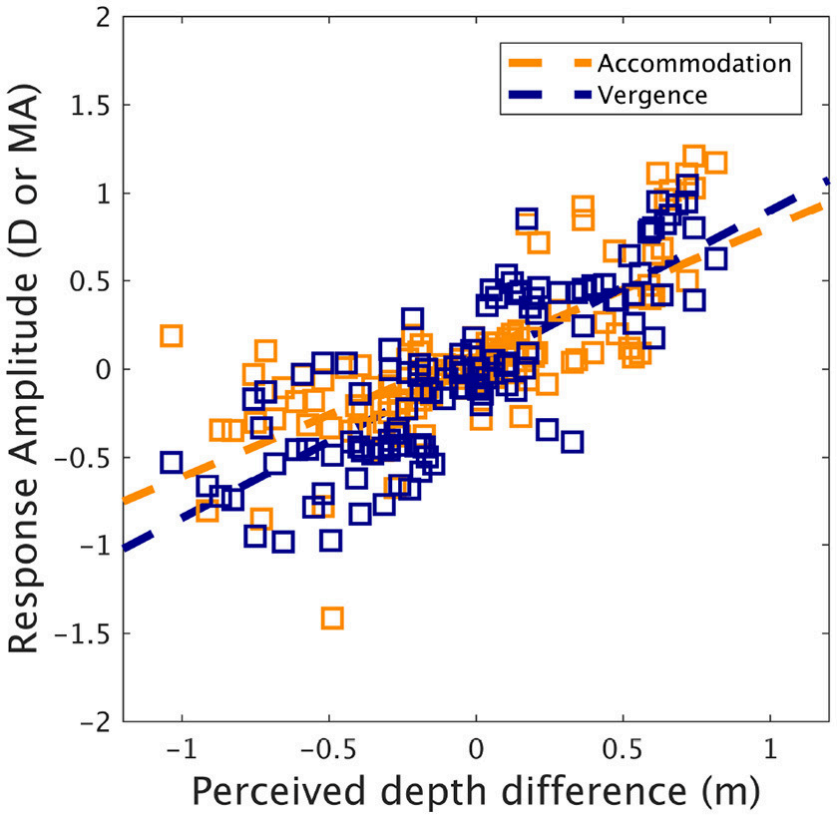
Accommodation and vergence response amplitude as a function of perceived depth difference (dark orange: accommodation, dark blue: vergence). Lines represent linear regression fit. Note that negative values refer to divergence or disaccommodation, while positive values refer to convergence or accommodation.

To understand how accommodation and vergence influenced perceived depth, scaling distances were converted into perceived depth changes by subtracting the accommodation distance (i.e., the screen plane distance) from scaling distances. This transform was performed because changes in vergence/accommodation from tonic states more likely contribute to perceived depth than absolute states (Von Hofsten, 1976). Correlations (see Figure 5) were obtained between the perceived depth difference and the accommodation response amplitude (Pearson: *R* = 0.71, *p* < 0.0001, Spearman: *R* = 0.78, *p* < 0.0001) and the vergence response amplitude (Pearson: R = 0.81, *p* < 0.0001, Spearman: R = 0.83, *p* < 0.0001). Next, we ran a multiple regression analysis on the perceived depth changes with accommodation amplitude and vergence amplitude as predictors. The multiple regression model was highly significant [*F*_(*F*(2, 114)_ = 117.9, *p* < 0.0001] and the R squared was equal to 0.67. The predictors accommodation amplitude and vergence amplitude were found to significantly affect perceived depth (*p* < 0.01, *p* < 0.0001, estimators coefficients were: 0.22 for accommodation and 0.59 for vergence). Figure 5 represents the relation between accommodation amplitude and perceived depth difference, and between vergence amplitude and perceived depth difference.

## 4. Discussion

A first result of this study is that depth constancy is affected by distance conflict between accommodation and vergence, mostly for a stimulus displayed behind the screen plane (i.e., negative steps). For example, stimulus depth is perceived to be flattened when its vergence distance is further than the focal distance. A second result is that both accommodation and vergence responses were correlated with perceptual estimates, thereby suggesting a plausible extra-retinal contribution. However, regression analyses revealed that vergence responses contributed more to explaining perceptual estimates of depth than accommodation responses.

A comparison of our results shows that distance conflict with fixed focal distances affects both perceptual estimates and accommodation response amplitudes for negative disparity steps, but not for positive steps. Vergence response amplitudes remained unchanged whatever the direction of the step stimulus. It is worth noting that this experiment did not display blur cues that could specify depth changes and, therefore, accommodation responses ensued from the vergence-accommodation link. In such a condition, Maiello et al. (2018) showed that accommodation responses have low gains when the stimulus is displayed away from the display surface. Our findings are consistent with these results but also indicate that accommodation gains are however fairly good for positive steps. Interestingly, when the accommodation response occurred with a reasonable gain (e.g., in response to a +1 D conflict), participants accurately perceived the depth of the stimulus. On the contrary, when the accommodation response did not occur or when its gain was low (e.g., in response to a 1 D conflict), whereas vergence gain was high, participants showed a bias in perceived depth. As disparity-driven accommodation stimuli were similar for negative and positive steps, perceived depth appears to have been affected by the accommodation response, as opposed to the accommodative stimulus. Therefore, these results strongly suggest that the accommodation response contributes to perceived depth and that its absence entails perceptual biases.

The reported correlations cannot, at first glance, be said to represent a causal effect. Nevertheless, previous studies have directly manipulated accommodation and vergence,and have shown consecutive effects on perceived depth, distance, and size (von Holst and Mittelstaedt, 1950; Brenner and Van Damme, 1998; Brenner and van Damme, 1999; Tresilian et al., 1999; Mon-Williams and Tresilian, 2000). Other interesting studies have explored the resting state hypothesis of accommodation (Owens and Liebowitz, 1980). When open-loop accommodation is tested using pinhole viewing, the system adopts a resting state (around 1D) that significantly affects distance judgments. Therefore, there is a consensus around the idea that perceived depth is affected by both the stimulus and responses of accommodation and vergence, which are themselves influenced by the stimulus.

If accommodation and vergence responses are sources of information for distance perception, they can indirectly affect perceived depth. Current models of cue combination assert that, because binocular disparities are ambiguous, they must necessarily be scaled using an estimate of distance (Landy et al., 1995). As this estimate of distance is influenced by the presence of a number of cues, perceived depth can be indirectly affected. Thus, to judge the depth of our stimulus, observers should have scaled the binocular disparity in the stimulus using an estimate of its egocentric distance (Bradshaw et al., 1996). This indirect effect on depth perception has already been observed in studies on the effect of focus cues on depth perception (Watt et al., 2005; Hoffman et al., 2008; Vienne et al., 2015). Our results thus suggest that bias in perceived depth indirectly ensues from undershoots in the disparity-driven accommodation response.

Two hypotheses can be advocated to explain how the accommodative response influences perceived depth. The first is the zoom-lens hypothesis (Roscoe, 1989), which describes a category of effects related to changes in the shape of the crystalline lens, and its effect on how light is cast onto the retina. This assumption is contrafactual, but let us examine the logic of its proposal. When an accommodation response is elicited, the shape of the lens changes which, in turn, changes the retinal image size. When defocusing from near to far (i.e., disaccommodating), the lens becomes flatter, and as a result, the retinal image size increases (Smith et al., 1992). If this small variation is noticed, it could be interpreted as a depth change. In our experiment, the simulation of vergence by binocular disparity could have produced a vergence accommodation response despite the absence of blur on the retinal image. In cases where there was no accommodation response, less depth change would have been mediated. Though interesting, the zoom-lens hypothesis is challenged by the fact that the magnitude of optical changes caused by accommodation appears to be so small that it cannot account for the effect size observed in accommodative micropsia (Smith et al., 1992).

A second hypothesis proposes that the contribution of accommodation is based on extra-retinal signals. Extra-retinal influences on position coding and localization have been demonstrated in a number of studies as afference from proprioception and efference copy of the oculomotor command (Bridgeman and Stark, 1991). As such, the capacity of performing step changes of accommodation to targets viewed through a pinhole pupil (McLin and Schor, 1988) suggests that visual feedback is not necessary for a step response, and that afference and/or efference are an effective signal for accommodation. Until recently, because ciliary bodies are smooth muscles, they were considered to have a poor neuromuscular innervation, suggesting that the idea that accommodation influences depth perception through the sensing of proprioceptive inflow might be mistaken. However, a recent study has revealed that proprioceptors are present in ciliary muscles and that they may serve a modulation role in the activity of the accommodation system (Flügel-Koch et al., 2009). Some authors have thus suggested that this proprioceptive input could provide extra-retinal signals which would contribute to estimate distances (Held et al., 2012). This is plausible but only if these nerve cells also include projections to higher level cellular organizations. Another option is to consider the neural commands to accommodate that are sent to ciliary bodies as possible influence in perceived size and distance (Brenner and van Damme, 1999; Mon-Williams and Tresilian, 2000). The idea that such a signal could account for the ability to discriminate retinal changes indicating movements in the external world from those arising from movements of the eyes by comparing efferent motor command with afferent feedback was proposed by Helmholtz (Von Helmholtz, 1867). In a similar vein, von Holst and Mittelstaedt (1950) proposed that accommodation micropsia (i.e., the apparent shrinkage of retinal image with accommodation) resulted from the influence of command signals from accommodation. In their experiment, the authors paralyzed accommodation using an atropine drug; when observers had to accommodate from far-to-near, they experienced micropsia as no afferent feedback was available, and the vergence stimulus was held constant (McCready and Donald, 1965). Such an efference copy of the accommodation command could thus also contribute to perceived distance and depth.

Because accommodation and vergence have different motor plants and specific neural pathways (Mays and Gamlin, 1995), it may be that each system response provides its own extra-retinal contribution based on oculomotor neural commands and/or proprioceptive afference. In a cue-poor environment, these extra-retinal cues could contribute to estimates of vergence and accommodation distance. These estimates could be integrated with other cues as proposed in current cue combination models (Landy et al., 1995). In a cue-rich environment, an interesting question is whether these extra-retinal contributions could still provide significant, relevant information for distance and depth perception. Complaints from users of stereoscopic displays, Virtual Reality (VR) or Augmented-Reality (AR) systems suggest that the issue may not be overcome for at least some individuals, given the wide inter-individual differences that are found when using these systems.

The findings of the present study have implications for the design of VR and AR systems, particularly for modern Head-Mounted Displays (HMD). A recent empirical work (Koulieris et al., 2017) has evaluated accommodation as well as visual discomfort in HMD, comparing three plausible solutions that should lessen the accommodation–vergence conflict, i.e., the major cause of visual discomfort. Instead of depth-of-field simulation and monocular viewing, using a focus-adjustable-lens system did improve comfort and enabled accommodation responses to simulated depth. Considering our result, we can predict that this setup should also improve depth perception because of a reduced accommodation-vergence conflict. Such focus-adjustable-lens systems thus may overcome discomfort as well as misperception of depth in VR setups given they allow a more natural correlation between vergence and accommodation responses.

## Ethics Statement

This study was carried out in accordance with the recommendations of the CEEI (Comité d'évaluation éthique de l'Inserm), Institutional Review Board (IRB00003888) of Inserm. All subjects gave written informed consent in accordance with the Declaration of Helsinki. The protocol was approved by the CEEI.

## Author Contributions

CV designed and performed the experiment, analyzed the data, performed the statistical analyses, created the graphics and wrote the paper. CV, JP, PN, and A-EP reviewed the paper and provided suggestions. All authors approved the final version of the manuscript for publication.

### Conflict of Interest Statement

The authors declare that the research was conducted in the absence of any commercial or financial relationships that could be construed as a potential conflict of interest.

## Figures and Tables

**Figure A1:**
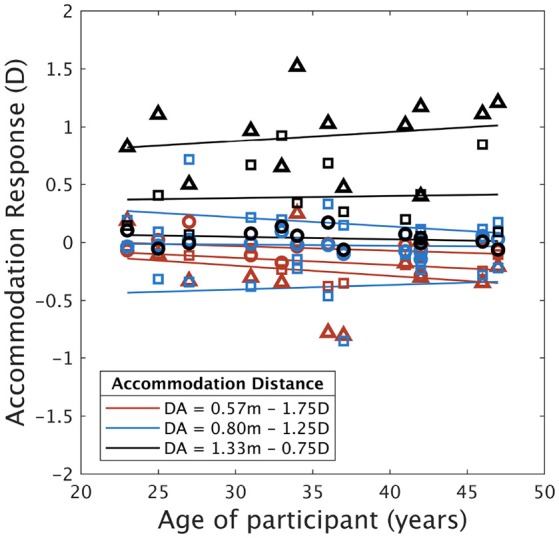
Accommodation responses as a function of participant's age (sorted in ascending order) for each accommodation distances (royal blue: 0.57 m, cadet blue: 0.80 m, and medium green: 1.33 m; conflict is represented by circles: 0 D, squares: ± 0.5 D, and triangles: ± 1 D). Lines represent linear regression fit for each set of distances. Negative values refer to disaccommodation, while positive values refer to accommodation.

## A. Appendix

The human visual function of accommodation declines with age, providing a reduced ability to focus at near distance, called presbyopia (Duane, 1922). However, this progressive deficit shows high interindividual differences. For example, Duane (1922) reported that 48 years-old people have an accommodation amplitude between about 1.4 and 4.5 Diopters and that, 42 years old people have near-point nearly between 2.5 and 7.2 D. Because of the near stimulus distance used in this study (a minimum of 1.75 D) and the relative old age of some participants, we address an analysis of accommodation responses as a function of participant age. Figure A1 represents the accommodation responses of the participants as a function of the accommodation-vergence distances and the age of the participants. To investigate a potential effect of age, we performed a three-way ANOVA on accommodation responses, including variable 'age' as grouping factor (individual below 40 years and individual beyond 40 years). The analysis revealed a significant effect of the accommodation and vergence distances on accommodation response [*F*_(2, 22)_ = 58.16, *p* < 0.0001; *F*_(2, 22)_ = 53.66, *p* < 0.0001, respectively], as well as an interaction effect between the two variables [*F*_(4, 44)_ = 9.24, *p* < 0.0001] but no effect of age [*F*_(1, 11)_ = 0.031, *p* > 0.05]. Because we cannot conclude from an absence of significant statistical difference, we performed a TOTS procedure (Lakens, 2017) to test for the equivalence of accommodation responses between the young participants and the older ones. A confidence interval of the mean difference in accommodation response between the two groups was computed. The mean difference was equal to 0.033 D (lower bound: –0.075 D - upper bound: 0.142 D). The CI was thus included in a ±0.15 D equivalence bounds, which can be reasonably considered as negligible effect size. The effect of age was thus non significant and the groups revealed equivalent accommodation responses. Additionally, we performed a linear regression on accommodation response over ages for each combination of accommodation-vergence distances. Slopes of linear regressions were all nearly equal to zero and varied from –0.009 to 0.008 D.year^−1^ for all combinations of accommodation and vergence distances [all linear regressions were non significant (*p* > 0.05), see Figure A1]. These results suggest that accommodation responses did not vary as a function of the age of the participants in the present study. We also computed correlations between accommodation response and age; none of them were significant (*p* > 0.05). Figure A1 reveals no apparent linear relationship between the two variables, and no variance appears to be explained in variation of accommodation response by changes in age.
